# How do autistic severity and family functioning influence parental stress in caregivers of children with autism spectrum disorder in China? The important role of parental self-efficacy

**DOI:** 10.3389/fpsyg.2023.956637

**Published:** 2023-05-25

**Authors:** Tomoko Kishimoto, Shuang Liu, Lumei Zhang, Shaowei Li

**Affiliations:** ^1^Department of Social Psychology, Zhou Enlai School of Government, Nankai University, Tianjin, China; ^2^CAS Key Laboratory of Behavioral Science, Institute of Psychology, Chinese Academy of Sciences, Beijing, China; ^3^Huli District Maternal and Child Health Hospital, Child Healthcare Department, Xiamen, China

**Keywords:** ASD, parental stress, parental self-efficacy, China, family adaptability and cohesion

## Abstract

**Introduction:**

Parental stress among primary caregivers of children with autism spectrum disorder (ASD) is a significant concern. While previous research indicates that both family and child factors substantially influence parental stress, a few studies have comprehensively examined these factors from family, parent, and child perspectives. Moreover, the psychological mechanisms underlying parental stress remain underexplored.

**Method:**

This study obtained a valid sample of 478 primary caregivers of children diagnosed with ASD in China and employed mediation and moderated mediation analyses to investigate the relationships between family adaptability and cohesion (FAC), ASD severity, parental self-efficacy, and parental stress.

**Result:**

Results revealed that higher FAC was linked to reduced parental stress through increased parental self-efficacy. The indirect effect of parental self-efficacy was more substantial for caregivers of children with severe symptoms than those with mild symptoms.

**Discussion:**

These findings offer insights into how FAC influences parental stress and underscore the importance of parental self-efficacy as a coping resource for mitigating parental stress. This study provides valuable theoretical and practical implications for understanding and addressing parental stress, particularly in families raising children with ASD.

## 1. Introduction

Caring for a child with autism spectrum disorder (ASD) can be highly stressful for parents, often more so than parenting children with other neurodevelopmental disabilities (Karst and van Hecke, 2012; DePape and Lindsay, 2014; Smith et al., 2014; Valicenti-Mcdermott et al., 2015; Pisula and Porebowicz-Dörsmann, 2017; Di Renzo et al., 2021). Elevated stress levels can negatively impact parents' mental health (Johnson et al., 2011) and subsequently affect the development of children affected by ASD, including communication, behavioral, and social issues (Hastings et al., 2005; Schieve et al., 2007), thus creating a vicious cycle. Therefore, identifying the factors influencing parental stress is crucial.

Numerous studies have explored various factors contributing to parental stress in families of children affected by ASD, including child-related factors such as hyperactivity (McStay et al., 2014a), irritability (Valicenti-Mcdermott et al., 2015), and IQ (Pastor-Cerezuela et al., 2016), as well as parent-related factors such as resilience, education, and socioeconomic background (Moh and Magiati, 2012; Pastor-Cerezuela et al., 2016). Environmental factors, like family resources (Derguy et al., 2016) and quality of life (Hsiao et al., 2017), have also been linked to parental stress. Some research has investigated predictors from both child and family perspectives, including problem behavior, symptom severity, family environment, and gender differences (McStay et al., 2014b). However, a few studies have proposed and tested comprehensive models integrating child, family environment, and individual parenting factors to explain parental stress in families with children affected by ASD. Therefore, this study aimed to address these gaps by exploring a reliable psychological mechanism behind parental stress in families of children with ASD using a large sample (*N* = 570). We seek to develop a model that considers multifaceted factors, including child aspects, family environment aspects, and individual parenting aspects. We also aimed to investigate the practical implications and intervenability of this model, as well as potential interactions between multiple factors, such as child and family environment influences on parents' cognition and psychological wellbeing.

Our central variables and hypothetical model draw from two theories: the double ABCX model [also called as family adjustment and adaptation response (FAAR) model; McCubbin and Patterson, 1983] in family psychology and the ecological systems theory (Bronfenbrenner, 1992) in developmental psychology.

The double ABCX model outlines critical factors influencing family adjustment and adaptation, where the stressor event (A) interacts with the family's crisis-meeting resources (B) and the family's perception of the event (C) to produce a crisis (X; McCubbin and Patterson, 1983). Though not specifically designed for explaining parental stress in families of children with ASD, it highlights the importance of family adaptive resources (B factor) in response to a crisis. Family functioning, as a family's crisis-meeting resource, including family adaptability and cohesion (FAC), plays a crucial role in the double ABCX model (Dabrowska and Pisula, 2010). Adaptability refers to the family's ability to change in response to stress, while cohesion is the emotional bonding among family members (Olson, 2000). Studies have linked high FAC levels with lower parental stress in various family situations (Gau et al., 2012; Dervishaliaj, 2013; León et al., 2015). We hypothesize that FAC negatively correlates with parental stress in families with ASD children.

Parental self-efficacy (PSE) is an important and modifiable factor in parental stress-related research for families of children affected by ASD (May et al., 2015; Li et al., 2022; Stephenson et al., 2022; Strauss et al., 2022). PSE has been linked to better stress management, less distress, and improved health outcomes (Taylor and Stanton, 2007) and has been found to significantly impact parental stress in parents of children affected by ASD (Boyraz and Sayger, 2011; Giallo et al., 2011; May et al., 2015; Li et al., 2022; Stephenson et al., 2022; Strauss et al., 2022). We hypothesize that PSE negatively correlates with parental stress in families with ASD children.

Although both FAC and PSE influence stress, a few studies have explored their relationship or established a pathway to explain the formation and alteration of parental stress related to ASD. We aimed to examine whether PSE mediates the relationship between FAC and parental stress, hypothesizing that FAC positively correlates with PSE and that FAC influences parental stress through PSE.

Following the ecological systems theory, we propose a larger statistical model incorporating a child-side factor as a moderator: children's ASD symptoms. We aimed to investigate whether the aforementioned mediation model holds under varying degrees of ASD symptom severity. Thus, our study explores a reliable psychological mechanism behind parental stress in families of children with ASD, integrating child, family, and parental factors into a moderated mediational model.

In this study, we aimed to investigate a reliable psychological mechanism behind parental stress in families of children with ASD using a large sample. Our goal was to integrate factors from the child, family, and parental perspectives to propose a moderated mediational model, offering a more comprehensive understanding of the psychological mechanisms underlying parental stress in caregivers of children with ASD. To date, there has been a scarcity of research exploring such mechanisms. This study seeks to provide insights into how parental stress can be influenced by child, family, and parental factors, and to identify potential effective interventions to enhance the psychological wellbeing of parents or caregivers of children with ASD.

## 2. Methods

### 2.1. Participants

Our participants were all primary caregivers (e.g., parents) of children affected by ASD from three rehabilitation institutions in Beijing, Tianjin, and Fujian. Data were collected by distributing an online questionnaire to the participants. Inclusion criteria were as follows: (1) primary caregivers; (2) having children diagnosed with and currently suffering from ASD; (3) children with ASD being no older than 18 years; (4) children with ASD receiving treatment in the contacted rehabilitation facilities; (5) Chinese; (6) able to use mobile phones and complete online questionnaires. Exclusion criteria were as follows: (1) inability to read and complete online questionnaires; (2) not having children diagnosed with ASD; (3) failure to pass the probe question filtering out careless responses; (4) inability to provide child's information, such as age; (5) children with ASD older than 18 years.

Data were collected from a total of 570 participants. Following Meade and Craig's (2012) recommendations for detecting careless responses, we excluded data with incorrect answers to the probe question (*N* = 92). The probe question read, “Please choose ‘Agree' for this question, or your data will be considered invalid.” We also excluded data with missing values (e.g., child's age, *N* = 23) and from participants whose children were older than 18 years (*N* = 16). The final sample consisted of 478 participants, comprising 25.94% fathers, 69.87% mothers, and 4.18% other relatives (e.g., grandparents). Detailed sample information can be found in Table 1.

**Table 1:** Socio-demographic characteristics of participants.

**Variables**	**Level**	***N***	**Proportion (%)**
**Participants (caregivers)**			
Relationship/gender	Father	124	25.94
	Mother	334	69.87
	Other	20	4.18
Age	Between 18 and 30	78	16.31
	Between 31 and 45	342	71.55
	Between 46 and 60	53	11.09
	Above 60	5	1.05
Employment status	Full-time	202	42.26
	Unemployed	178	37.24
	Part-time	52	10.88
	Other	46	9.62
Household income	Below 5,000	110	23.01
	Between 5,001 and 10,000	156	32.64
	Between 10,001 and 20,000	139	29.08
	Above 20,000	73	15.27
Marital status	Unmarried	12	2.51
	Married	413	86.40
	Divorced	21	4.39
	Widowed	25	5.23
	Remarried	5	1.05
	Other	2	0.42
**Autism patients (children)**			
Gender	Male	375	78.45
	Female	103	21.55
Age	5.43 ± 3.50 (Mean); Max = 18, Min = 0	478	\
Severity	Mild	253	52.93
	Moderate	180	37.66
	Severe	45	9.41
Comorbidity	Intellectual disability	60	12.55
	Delayed language development	196	41.00
	ADHD	66	13.81
	Sensory disorders	12	2.51
	Cerebral palsy	6	1.26
	Epilepsy	25	5.23
	Other	27	5.65
	Not suffered any	86	17.99

ADHD, attention deficit hyperactivity disorder.

### 2.2. Measurements

#### 2.2.1. Parental stress

We used the Chinese version of the Parenting Stress Index/Short Form, PSI-SF (Abidin, 1995). It has a total of 36 questions, including three subscales: parental distress (PD), parent–child dysfunctional interaction (PCDI), and difficult child (DC). Participants were asked to report their feelings on a 5-point scale. The higher the score, the higher level of parental stress experienced. The total score of this scale ranged from 36 to 180 points: 90 points are the critical level of parental stress in clinical significance, 91–98 points may indicate high levels of parental stress, and 99 points or above may indicate extremely high levels of parental stress (Wang et al., 2013). A Cronbach's α coefficient from a recent peer-reviewed study in China is 0.79, based on a sample size of 479 (Lu et al., 2018). The Cronbach's α coefficient in this study was 0.95.

#### 2.2.2. Family adaptability and cohesion

We used Family Adaptability and Cohesion Evaluation Scales II, Second Edition-Chinese Version (FACES II-CV; Olson et al., 1979; Fei et al., 1991) (Olson et al., 1979). It has a total of 30 items, measuring family adaptability (14 items) and family cohesion (16 items) separately. The adaptability subscale measures the abilities of a family to change in a stressful situation, like “our family tries new ways of dealing with problems;” and the cohesion subscale measures the bonding and supportiveness of a family, like “family members are supportive of each other during difficult times.” Participants need to report the extent to which the situation happens in their family life according to the reality from 1 (none at all) to 5 (always). We included both of the total scores and analyze the models for them separately according to previous studies (e.g., Glenn et al., 2009; Boyraz and Sayger, 2011). A higher score indicates a higher level of adaptability/cohesion. The Cronbach's α coefficients in the original Chinese version are 0.73 (adaptability) and 0.85 (cohesion; Fei et al., 1991). The Cronbach's α coefficients in the current study were 0.88 (adaptability) and 0.85 (cohesion). Family adaptability and cohesion are positively correlated (*r* = 0.83, *p* < 0.001).

#### 2.2.3. Parental efficacy

To measure the perception of parenting competence or efficacy, we adopted a widely used The Parenting Sense of Competence Scale (PSOC; Gibaud-Wallston, 1978). The Chinese version we used was composed of 12 items (Peng et al., 2012), assessing competence and satisfaction using a 4-point scale. The total score was used. The version we used is adapted from the original PSOC, and it deleted five items of the original 17-item scale according to the factorial structure of PSOC in China (Peng et al., 2012). For example, Items 1 and 5 in the original scale were deleted for a lower factor loading (below 0.3), and Items 12, 14, and 17 were deleted because of the inconsistency of cross-factor loading in the fathers' sample and mothers' sample. A higher score indicates higher levels of parental self-efficacy. The Cronbach's α coefficient in the original Chinese version sample was 0.85 (Ngai et al., 2007). The Cronbach's α coefficient in the present study was 0.62.

#### 2.2.4. Severity of autistic symptoms

We collected the severity of ASD-related symptoms from the main caregivers. The diagnoses were provided by psychiatrists and were based on DSM-5. The standard divides children with ASD into three severity levels based on impairment in social communication and restriction/repetitive behaviors. This study's mild, moderate, and severe levels correspond to the “need support,” “need substantial support,” and “need very substantial support,” respectively, in DSM-V (American Psychiatric Association, 2013). See Table 1 for this study's distribution of children's ASD severity.

#### 2.2.5. Demographics and other covariates

The demographic part consisted of information about the participants (main caregivers) and their children (diagnosed with autism). The age, household income per month, educational level, marital status, employment status of main caregivers, relation with the child, and children's gender, age, comorbidity, and symptom severity were collected. See Table 1 for detailed information on the sample.

### 2.3. Analysis procedure

Before our main analysis, we did a preliminary analysis: the descriptive information of our data; a correlational analysis for key variables (including parental stress and its possible factors: FAC, PSE, and ASD symptom severity); and a multivariate regression to show the simple effects of key variables in predicting parental stress. Then, we started our main hypothesis testing: testing for simple mediation models and the full moderated mediation model. First, we tested the indirect effect of FAC through PSE in predicting parental stress, i.e., the mediation role of PSE. We reported the two simple mediation models involving family adaptability and cohesion separately to reveal more information from the whole construct of FAC. The bootstrapped simple mediation modeling using 5,000 bias-corrected bootstrap samples by PROCESS macro version 3.5.2 (Model 4, Hayes, 2017) was used. All variables were standardized and centered before the mediation analysis. Next, when the simple mediation was supported, we examined the full hypothetical model—a moderated mediation model by considering the moderating role of the child's ASD symptom severity. Before the modeling process, we dummy-coded the three-level severity of symptoms by setting mild symptoms as the reference level, according to the method for examining the relative mediation effect (Hayes and Preacher, 2014). Then, we tested the moderated mediation models by PROCESS with 5,000 bias-corrected bootstrap samples (Model 7, Hayes, 2017).

## 3. Results

### 3.1. Preliminary analysis

The descriptive and correlation results were presented in Table 2. In general, parental stress was negatively correlated with FAC [*r*_(476) adaptability_ = −0.09, *p* < 0.001, *r*_(476) cohesion_ = −0.22, *p* < 0.001], PSE [*r*_(476)_ = −0.44, *p* < 0.001], and positively correlated with the severity of ASD-related symptoms [*r*_(476)_ = 0.13, *p* < 0.01]. Intercorrelations among the factors showed that greater family adaptability and cohesion both were associated with less severe ASD symptoms [*r*_(476) adaptability_ = −0.15, *p* < 0.001; *r*_(476) cohesion_ = −0.19, *p* < 0.001] and a higher level of PSE [*r*_(476) adaptability_ = −0.33, *p* < 0.01; *r*_(476) cohesion_ = 0.30, *p* < 0.001] and was then related to a lower level of parental stress.

**Table 2:** Descriptive statistics and correlations.

**Variables**	**Mean**	**SD**	**1**	**2**	**3**	**4**	**5**
1. Parental stress	106.29	24.02	\				
2. Adaptability	48.09	9.08	−0.09^*^				
3. Cohesion	55.50	10.40	−0.22^***^	0.83^**^	\		
4. PSE	30.40	3.85	−0.44^***^	0.33^**^	0.30^***^	\	
5. Severity^a^	N/A	N/A	0.13^**^	−0.15^**^	−0.19^**^	−0.10^*^	\

N = 478. PSE, parental self-efficacy. N/A, not applicable.

^*^p < 0.05.

^**^p < 0.01.

^***^p < 0.001.

^a^Spearman correlation was used when including the severity factor.

The multivariate regression analysis showed the effect of key variables (adaptability, cohesion, and PSE) was strong than other factors (see Table 3).

**Table 3:** Multivariate regression predicting parental stress.

**Predictor**	***B***	**SE**	***t***	***P***
Intercept^a^	0.21	0.32	0.67	0.50
**Key variables**				
Adaptability	0.35	0.07	4.70	< 0.001
Cohesion	−0.33	0.07	−4.40	< 0.001
PSE	−0.48	0.04	−10.81	< 0.001
**Other variables**				
Gender (reference: other)				
Father	−0.04	0.23	−0.19	0.85
Mother	−0.14	0.22	−0.65	0.52
Age (reference: 18–30 years old)				
31–45 years old	0.06	0.11	0.51	0.61
46–60 years old	−0.04	0.20	−0.20	0.84
Above 60	1.10	0.42	2.59	0.01
Job (reference: full-time)				
Unemployment	0.15	0.14	1.08	0.28
Part-time	0.12	0.10	1.24	0.22
Other	0.02	0.19	0.11	0.92
Income (reference: below 5,000)				
5,001–10,000	0.03	0.11	0.26	0.80
10,001–20,000	−0.23	0.11	−2.04	0.04
Above 20,000	−0.36	0.14	−2.61	0.01
Marriage (reference: unmarried)				
Married	−0.33	0.27	−1.23	0.22
Divorced	0.03	0.32	0.10	0.92
Widowed	−0.03	0.42	−0.08	0.94
Remarried	−0.20	0.28	−0.41	0.68
Other	0.07	0.65	0.11	0.92
Child Gender (reference: male)				
Female	0.08	0.10	0.82	0.41
Child age	0.11	0.05	2.25	0.03
Severity (reference: mild)				
Moderate	−0.09	0.09	−0.97	0.33
Severe	−0.08	0.14	−0.58	0.57
Comorbidity (reference: not suffered any)				
Intellectual disability	0.35	0.15	2.39	0.02
Delayed language development	0.17	0.11	1.55	0.12
ADHD	0.39	0.15	2.72	0.01
Sensory disorders	0.61	0.27	2.29	0.02
Cerebral palsy	0.19	0.37	0.50	0.61
Epilepsy	0.38	0.32	1.19	0.24
Other	−0.03	0.19	−0.19	0.85

^a^Represents reference level.

### 3.2. Simple mediation analysis

**Figure 1:**
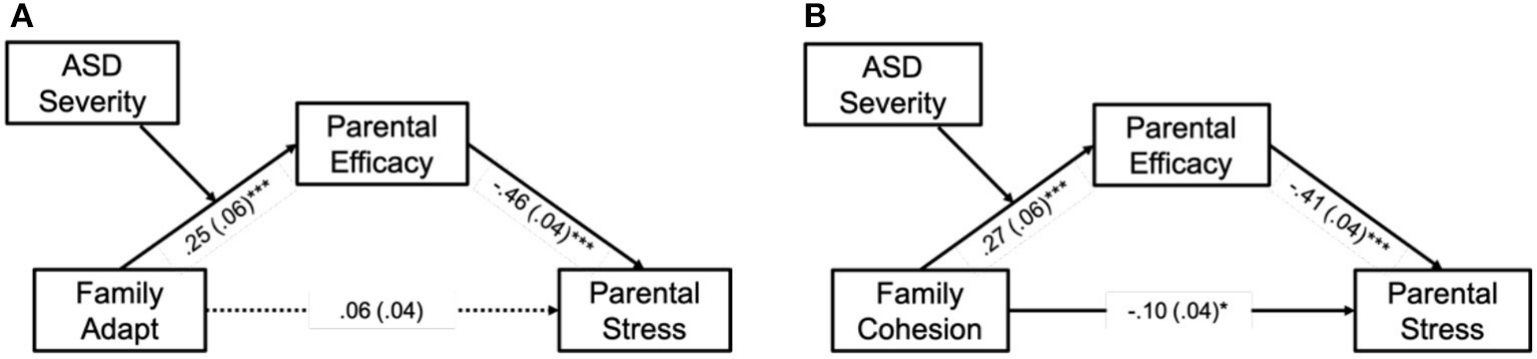
Moderated mediation models predicting parental stress. Family adapt, family adaptability. The figure shows the moderated mediation model in which the severity of children's autistic symptoms moderates the relationship between family adaptability/cohesion and parental self-efficacy and finally affects parental stress. **(A, B)** The models of adaptability and cohesion, respectively. Standardized regression coefficients (β) are presented above the arrows. Solid lines represent significant paths. Statistical significance: ^*^p < 0.05; ^***^p < 0.001.

The resulting models were significant and explained 9% (IV: adaptability) and 20.05% (IV: cohesion) of the total variance in parental stress. When adaptability was IV, the result showed a significant indirect effect through PSE when predicting parental stress [*B* = −0.15, 95% CI = (−0.1969, −0.1032)], but the direct effect was not significant [*B* = 0.06, 95% CI = (−0.0286, 0.1429)]. It suggested a fully mediating role of PSE in the model. When cohesion was IV, the result showed a significant indirect effect too [*B* = −0.12, 95% CI = (−0.1667, −0.0792)] and also a significant direct effect between family cohesion and parental stress [*B* = −0.10, 95% CI = (−0.1856, −0.0168)]—indicating a partial mediating role of PSE in this model. Taken together, the mediation models were supported. PSE was a full mediator in the relationship between adaptability and parental stress (Figure 1A) and a partial mediator between cohesion and parental stress (Figure 1B).

### 3.3. Moderated mediation analysis

The results showed that the interactions of severe (vs. mild) symptoms and FAC were significant in affecting PSE, but the interactions of moderate (vs. mild) symptoms and FAC were insignificant (see Table 4). Conditional effects of the severity of autistic symptoms showed that, under each severity level, the moderations were all significant in both moderation models, in which severe symptoms had the greatest effect. It indicated that different levels of autistic symptoms moderated the relationships between family adaptability/cohesion and PSE. At a severe level, FAC has a greater impact on PSE (see Table 5).

**Table 4:** Moderated mediation model predicting parental stress.

	***B***	***SE***	***t***	***P***	**95% CI**	***R* ^2^**	***F***
**Independent variable: family adaptability**							
**Moderation analysis**							
**Outcome variable: parental self-efficacy**						0.14	15.67
Family adaptability	0.25	0.06	4.15	< 0.001	[0.1317, 0.3690]		
Moderate (vs. mild)	−0.09	0.09	−1.01	0.32	[−0.2724, 0.0879]		
Severe (vs. mild)	−0.10	0.16	−0.64	0.52	[−0.4142, 0.2102]		
Adaptability × moderate	−0.01	0.09	−0.07	0.94	[−0.1911, 0.1776]		
Adaptability × severe	0.54	0.14	3.89	< 0.001	[0.2682, 0.8152]	0.03^***^	8.30
**Moderated mediation analysis**							
**Outcome variable: parental stress**						0.19	57.18
Family adaptability	0.06	0.04	1.31	0.19	[−0.0286, 0.1429]		
Parental self-efficacy	−0.46	0.04	−10.46	< 0.001	[−0.5411, −0.3700]		
**Independent variable: family cohesion**							
**Moderation analysis**							
**Outcome variable: parental self-efficacy**						0.12	12.40
Family cohesion	0.27	0.06	4.31	< 0.001	[0.1464, 0.3920]		
Moderate (vs. mild)	−0.08	0.09	−0.84	0.40	[−0.2629, 0.1057]		
Severe (vs. mild)	−0.17	0.16	−1.05	0.29	[−0.4818, 0.1454]		
Cohesion × moderate	−0.10	0.10	−1.01	0.31	[−0.2859, 0.0915]		
Cohesion × severe	0.37	0.14	2.75	< 0.01	[0.1061, 0.6392]	0.02^**^	5.63
**Moderated mediation analysis**							
**Outcome variable: parental stress**						0.20	59.55
Family cohesion	−0.10	0.04	−2.36	0.02	[−0.1856, −0.0168]		
Parental self-efficacy	−0.41	0.04	−9.48	< 0.001	[−0.4913, −0.3225]		

95% CI, 95% confidence interval. The severity of children's autistic symptoms was a categorical variable and thus was dummy-coded by setting mild symptoms as the reference level. Bootstrap sample size = 5,000.

**Table 5:** Conditional effects of severity of ASD symptoms.

**Severity level**	**Effect**	***SE***	**LLCI**	**ULCI**
**IV: family adaptability**				
**Conditional effects (predicting parental self-efficacy)**				
Mild	0.25	0.06	0.1317	0.3690
Moderate	0.24	0.07	0.1025	0.3846
Severe	0.79	0.13	0.5456	1.0384
**Conditional indirect effects (predicting parental stress through**				
**parental self-efficacy)**				
Mild	−0.11	0.03	−0.1690	−0.0608
Moderate	−0.11	0.03	−0.1782	−0.0499
Severe	−0.36	0.09	−0.5327	−0.1966
**Difference between conditional indirect effects**				
Moderate—mild	0.01	0.04	−0.0760	0.0816
Severe—mild	−0.25	0.09	−0.4197	−0.0814
**IV: family cohesion**				
**Conditional effects (predicting parental self-efficacy**				
Mild	0.27	0.06	0.1464	0.3920
Moderate	0.17	0.07	0.0288	0.3152
Severe	0.64	0.12	0.4054	0.8784
**Conditional indirect effects (predicting parental stress through**				
**parental self-efficacy)**				
Mild	−0.11	0.03	−0.1683	−0.0564
Moderate	−0.07	0.03	−0.1308	−0.0125
Severe	−0.26	0.07	−0.4029	−0.1262
**Difference between conditional indirect effects**				
Moderate—mild	0.04	0.04	−0.04	0.12
Severe—mild	−0.15	0.07	−0.30	−0.02

LLCI, lower limit confidence intervals; ULCI, upper limit confidence intervals.

For the whole mediation model that continued to predict parental stress, we found that across all levels of severity, the indirect effects of PSE on the relationship between FAC and parental stress were significant no matter when the independent variable was family adaptability or cohesion (see Table 4). Nevertheless, the analysis of the difference between conditional indirect effects showed that only for caregivers of children with severe (vs. mild) symptoms rather than moderate (vs. mild) symptoms, the degree of reducing parental stress through the indirect pathways was significant and greater (see Table 5).

Therefore, the moderated mediations were supported in that severity of autistic symptoms moderated between family adaptability/cohesion and PSE and then influenced parental stress. Compared with mild symptoms, the degree of reducing parental stress through indirect pathways is greater in the condition of severe symptoms.

## 4. Discussion

The formation of parental stress when raising children with autism spectrum disorder (ASD) symptoms has been recognized by theories and evidence to be critical to factors in the family and within the parent domain (McCubbin and Patterson, 1983; Abidin, 1992; Hastings et al., 2005; McStay et al., 2014b; Rivard et al., 2014; Brei et al., 2015; Porter and Loveland, 2019). However, insufficient attention has been paid to the underlying mechanism, and research on this process that integrates factors from family, parents, and children together is limited. To explore a reliable psychological mechanism behind parental stress, this study collected data from a large sample of primary caregivers who have children affected by ASD in China and performed a moderated mediation model to examine the hypothesis. The results of this study confirm that parental self-efficacy can alleviate the relationship between family dysfunction and parental stress, supporting the first hypothesis. In families with children affected by ASD, family dysfunction can lead to parental stress. For instance, research on families with children affected by ASD found that family function has a significant effect on maternal parental stress, while couple adjustment has a specific effect on paternal parental distress (Di Renzo et al., 2021). Therefore, researchers must pay attention to both the mitigative and protective factors of family dysfunction on the stress of primary caregivers of children affected by ASD. Understanding the potential factors contributing to the association between family dysfunction and parental stress may provide empirical evidence for the development of intervention strategies and psychological health services for relevant populations.

Parental self-efficacy is a crucial factor in child-rearing that can have a simultaneous impact on the behavior of parents and the development of children. Parents' ability to discriminate education, communicate, and exhibit behavioral tendencies are perceived and influenced by their level of parental self-efficacy (Jones and Prinz, 2005). If parents can enhance their parental self-efficacy, they will have more confidence, be more active in communicating and interacting with their children, and adopt better educational behaviors, positively affecting children's cognitive, emotional, and behavioral development. In previous studies, parental self-efficacy in families with children affected by ASD was found to be moderately correlated with parental stress (Batool and Khurshid, 2015), consistent with the findings of this study. Furthermore, research has shown that parental self-efficacy can alleviate the negative impact of a lack of adaptive resources and increase an individual's level of adaptability. For example, a study on 10,947 Chinese families found that parental self-efficacy mediated the effect of social support on parenting stress (Hong and Liu, 2021). However, no empirical research has verified this effect in families with children affected by ASD, except for one study that attempted to verify the interaction effect of family resources and parental self-efficacy on parental stress in families with children affected by ASD. After controlling for symptom severity, the study did not find a significant interaction effect (Stephenson et al., 2022), suggesting that there may be other explanatory models.

This study examined two models of family adaptability and cohesion separately, and the evidence suggests that poorer family adaptability can lead to higher levels of parental stress, a process fully mediated by parental self-efficacy. Parental self-efficacy is an essential protective factor in the process of parental stress caused by poorer family adaptability. Family cohesion is partially mediated by parental self-efficacy, indicating that parental self-efficacy can partly alleviate parental stress caused by insufficient interdependence, close connections, and emotional fusion among family members. The difference between adaptability and cohesion may be due to the fact that self-efficacy is more focused on an individual's agency and control in achieving their goals. Thus, building self-efficacy may have a greater influence in helping develop adaptability and resilience in the face of change and adversity. In summary, this study's evidence suggests that promoting parental self-efficacy among primary caregivers of children affected by ASD is crucial for reducing parental stress, especially for reducing the stress caused by family dysfunction.

The study's results also show that the severity of children's autistic symptoms moderates the relationship between family adaptation and cohesion and parental self-efficacy, which then influences parental stress. The more severe the symptoms, the stronger the negative impact of family dysfunction on parental stress. Individuals with higher levels of family dysfunction are more likely to report severer parental stress, often due to the severer symptoms of children. These findings are consistent with previous research on the correlation of children's symptom severity with family dysfunction and parental stress (Hastings and Johnson, 2001; Kogan et al., 2008; Estes et al., 2013). Furthermore, the study's findings indicate that different levels of severity in children's autistic symptoms can moderate the relationship between FAC and PSE, which then influences parental stress. In cases of severe symptoms, the study found the strongest association between FAC and PSE, which indicates that caregivers with children who have severe autistic symptoms need to pay more attention to the family environment and enhance their self-efficacy. However, when including all the factors together, the study did not find a significant effect from the severity of symptoms, even though some previous studies suggest it is a significant factor (Hastings and Johnson, 2001; Tobing and Glenwick, 2002; Baker-Ericzen et al., 2005; Benson, 2006; Ingersoll and Hambrick, 2011; Rivard et al., 2014). This finding suggests that the severity of symptoms is relatively less important than FAC and PSE in affecting parental stress.

To summarize, this study emphasizes the significance of intervenable factors from the parent's perspective and underscores the crucial role of parental self-efficacy in reducing parental stress. The study reveals that parental self-efficacy not only positively impacts the reduction of parental stress but also acts as an essential partial mediator between family cohesion and parental stress and a full mediator between family adaptability and parental stress. Additionally, regardless of the varying degrees of ASD symptom severity, parental self-efficacy always has a robust mitigating effect on parental stress, with an even more potent effect in cases of severe ASD symptoms.

### 4.1. Implications

This study has practical implications for intervening in parental stress experienced by caregivers with children diagnosed with ASD. First, given the significance of family adaptability and cohesion, it is necessary to provide family interventions and psychotherapeutic treatments for children displaying ASD symptoms. To alleviate parental stress in families with children exhibiting severe ASD symptoms, it is particularly important to focus on interventions targeting family adaptability and cohesion. For instance, Multisystemic Family Intervention is a family-centered therapy that explores the interactions between children and family members, identifies factors within the family that may affect the child's behavior and adaptation, emphasizes cooperation and communication among family members to improve family adaptability and cohesion, and better support the development of children affected by autism (Sheidow et al., 2014). Second, and more importantly, parental self-efficacy plays a crucial mediating role between FAC and parental stress, particularly in conditions of severe symptoms. Prior research suggests that intervention programs that focus on collaboration, empowerment, and supporting families can indirectly enhance parental self-efficacy, such as family-centered practice (Hughes-Scholes and Gavidia-Payne, 2019). Other interventions specifically target parental self-efficacy for parents of children with ASD, such as Parent Management Training, which emphasizes helping parents develop skills in situational awareness, self-regulation, and goal-setting to enhance their self-efficacy (Sofronoff and Farbotko, 2002). The Confident Parents Program, based on Bandura's social learning theory, focuses on utilizing a combination of self-reflection, sharing and learning from parenting experiences, feedback, and practicing problem-solving and action planning to help parents better recognize their strengths and abilities, strengthen interactions and support among parents, and enhance their confidence and sense of self-efficacy (Mouton et al., 2018). Therefore, based on the findings of this study, it is recommended to implement interventions and training programs that incorporate the aforementioned elements for caregivers. These interventions can be tailored to meet the specific needs and circumstances of each family and aimed to enhance parental self-efficacy, improve family adaptability and cohesion, and reduce parental stress associated with caring for children diagnosed with ASD. Additionally, it is crucial to regularly assess the effectiveness of these interventions and make necessary adjustments to ensure that they continue to meet the needs of caregivers and families.

### 4.2. Limitations

While this study has yielded significant findings, it is not without limitations. First, while we have investigated the mechanism of parental stress from the family, parents, and child perspectives, we have not included other important factors that may contribute to parental stress, such as child IQ (Pastor-Cerezuela et al., 2016) and behavioral problems (Cooley et al., 2014; Robinson and Neece, 2015; Shawler and Sullivan, 2015; Chan and Lam, 2017). Future studies should consider these factors more comprehensively. Second, due to the cross-sectional nature of our study, we cannot establish a causal relationship through our simple mediation and moderated mediation results. We recommend that future studies use longitudinal data to verify our findings. Third, the sample in the current study was drawn from a limited number of provinces/municipalities in China, which may not fully represent the situation in China or other countries and cultures. Therefore, a large-scale research is needed to validate our hypotheses. Finally, while we found family adaptability and cohesion to be beneficial, other researchers have suggested that both higher and lower levels of family adaptability and cohesion may be associated with dysfunctional family interaction (Higgins et al., 2005). Therefore, the effect of family cohesion should be approached with caution, and future studies can explore key moderators in the relationship to supplement our models.

## 5. Conclusion

Through our survey of primary caregivers of children with ASD, we have identified several factors that can influence parental stress. We found that greater family adaptability and cohesion (FAC) was associated with lower levels of parental stress, and this relationship was mediated by parental self-efficacy (PSE). In addition, the beneficial effect of FAC through the indirect effect of PSE was found to be greater in families with children who had severe symptoms, than those with mild symptoms. These findings provide a deeper understanding of the complex interactions between family and child factors and their impact on parental stress, highlighting the importance of addressing the personal factors of caregivers in clinical interventions aimed at improving the mental health of parents of children with ASD.

## Data availability statement

The raw data supporting the conclusions of this article will be made available by the authors, without undue reservation.

## Ethics statement

The studies involving human participants were reviewed and approved by the Ethics Committee of Nankai University. The patients/participants provided their written informed consent to participate in this study.

## Author contributions

TK: conceptualization, methodology, data analysis, writing—reviewing and editing, and supervision. SLiu: writing—original draft preparation. LZ: conceptualization and investigation. SLi: resources and investigation. All authors contributed to the article and approved the submitted version.
